# Single Nano-Sized Metal–Organic Framework for Bio-Nanoarchitectonics with In Vivo Fluorescence Imaging and Chemo-Photodynamic Therapy

**DOI:** 10.3390/nano12020287

**Published:** 2022-01-17

**Authors:** Yong-Mei Wang, Ying Xu, Xinxin Zhang, Yifan Cui, Qingquan Liang, Cunshun Liu, Xinan Wang, Shuqi Wu, Rusen Yang

**Affiliations:** 1 School of Advanced Materials and Nanotechnology, Academy of Advanced Interdisciplinary Research, Xidian University, Xi’an 710126, China; wangym1001@xidian.edu.cn (Y.-M.W.); 18141213000@stu.xidian.edu.cn (Y.X.); xxinzhang20@stu.xidian.edu.cn (X.Z.); 17170210019@stu.xidian.edu.cn (Y.C.); 21010540018@stu.xidian.edu.cn (Q.L.); 21141213493@stu.xidian.edu.cn (C.L.); wangxn@xidian.edu.cn (X.W.); 2 School of Life Sciences, Northwest University of Technology, Xi’an 710072, China; sqwu@nwpu.edu.cn

**Keywords:** metal–organic framework (MOF), multifunction, fluorescence, chemotherapy, photodynamic therapy

## Abstract

Theranostics is an emerging technique for cancer treatments due to its safety and high efficiency. However, the stability, efficiency, and convenience of preparation are the main challenges for developing theranostics. Here we describe a one-pot process for biocompatible metal–organic framework (MOF)-based theranostics. The ligand H_2_L designed for the MOF enables both red fluorescence emission and photodynamic therapy (PDT). The frame and regular channel structure of H_2_L-MOF empower the theranostics with good drug delivery performance, and the uniform and nano-sized particles facilitate the in vivo imaging/therapy applications. In vivo fluorescence imaging and in vitro chemo-photodynamic therapy were achieved with the MOF without any further modification. Our results reveal an effective strategy to achieve multifunctional theranostics by the synergistic action of the organic ligand, metal node, and channel structure of MOF nanoparticles.

## 1. Introduction

Cancer is still a major disease that seriously imperils human life and health. The aging of a population, smoking, infection, and environmental pollution worsen the situation [1]. Chemotherapy, radiotherapy, and surgery are still the main treatments for cancer, notwithstanding their inevitable adverse effects [2,3]. The rapid and accurate diagnosis and effective treatment methods of tumors are still lacking and need to be developed.

The integration of both multimodal imaging agents and multiple therapeutic agents into theranostics is highly attractive and has been extensively studied for clinical applications. Theranostics uses imaging information to achieve time- and position-resolved therapy for a targeted tumor [4,5,6]. The key to this newly developed noninvasive diagnostic and therapeutic method is the preparation of probes with good imaging and therapy properties. Current research involves various strategies to achieve multi-functional theranostics probes, such as dopant modification [7], post-modification by subsequent chemical grafting [8], entrapping functional molecules in porous materials [9,10], and so on. For example, Luo et al. developed a core-shell nanomaterial-based all-in-one nanoplatform for in situ tumor imaging and simultaneous photothermal therapy [11]. However, the platform involves the preparation of nanobipyramids@polydopamine, gold clusters, and the composite of the two kinds of materials, which needs a complex preparation process and is time consuming. Meanwhile, as a commercialized anti-cancer drug, DOX has been widely used as a drug model to evaluate various kinds of nanocarriers’ delivery and release effects [4,12,13]. Liu et al. developed the CuS_NC_@DOX@MnO_2-NS_ nanoplatform for multi-mode imaging, chemical therapy, and photothermal therapy through layer-by-layer coating [14]. However, the current nanocarriers often involve complicated preparation processes for multifunctional probe materials, and the structural instability of the reported nanostructures is often a great concern.

Metal–organic frameworks (MOFs) are considered a promising material for theranostics, and are constructed from metal ions bridged by organic ligands and have a variety of structural types, low density, permanent pores, and ultra-high specific surface [15,16]. Their physical, chemical, and electrical properties can be easily tuned by incorporating different metal ions and organic ligands [17]. Nanocrystallization further expanded the applications in many fields, such as catalysis [18,19], gas storage and separation [20], sensing [21,22], bioimaging and therapy [23,24,25], and drug delivery [26,27,28]. The versatility of MOFs makes it possible to integrate multimodal imaging capability and target therapy functions in one single material by choosing appropriate building units. Chen et al. prepared Ru(bpy)_3_^2+^-incorporated UiO-67 NMOFs, which realized two-photon fluorescence imaging and photodynamic therapy [29]. It is a good method to realize multi-functional integration by constructing composite materials, but it involves complicated preparation processes and instability.

Herein, we propose a simple and cost-effective way to prepare multifunctional MOF nanoparticles for theranostics. Bis (2, 2′-bipyridy) (5, 5′-di-p-benzoicacid-[2, 2′] bipyridinyl) ruthenium (Ⅱ) dichloride (H_2_L) has the property of NIR-I fluorescence emission and singlet oxygen generation and is chosen as the organic ligand for the MOF. With biocompatible zirconium (Zr^4+^) ions as the metal node, we constructed the porous and multifunctional H_2_L-MOFs with a one-pot procedure and achieved nanoparticles that integrate NIR-I fluorescence imaging, photodynamic therapy, and drug delivery function in one material. The uniform H_2_L-MOF nanoparticles show a spherical shape, an average size of 50 nm, and good dispersion in water. We verified the excellent biocompatibility of H_2_L-MOF by conducting cell experiments and in vivo safety evaluations of mice. In vivo red fluorescence imaging of both normal mice and tumor-bearing mice showed rapid metabolism through the liver and targeted tumor. Chemotherapy, photodynamic therapy, and chemo-photodynamic therapy were demonstrated by cell experiments.

## 2. Materials and Methods

### 2.1. Materials

Zirconium tetrachloride (ZrCl_4_) and (9,10-Anthracenediyl-bis(methylene)-dimalonic acid (ABDA) were both purchased from Aladdin Biochemical Technology Co., Ltd. (Shanghai, China). Bis (2, 2′- bipyridy) (5, 5′-Di-p-benzoicacid-[2, 2′] bipyridiny) ruthenium (Ⅱ) dichloride (H_2_L) was purchased from SunaTech Inc. (Suzhou, China). N, and N-dimethyl formamide (DMF) and dimethyl sulfoxide (DMSO) were both purchased from Tianjin Fuyu Fine Chemical Co., Ltd. (Tianjin, China). Trifluoroacetic acid (TFA) was purchased from Tianjin Kemeiou Chemical Reagent Co., Ltd. (Tianjin, China). DOX•HCl was purchased from Beijing Huafeng Lianbo Chemical Materials Co., Ltd. (Beijing, China). 3-(4, 5-dimethythiazole-2-yl)-2, 5-phenyl tetrazolium bromide (MTT) was purchased from Jiangsu Keygen Biotech Co., Ltd. (Nanjing, China). All reagents were used as received without further purification.

### 2.2. Characterized Techniques

The morphology and elemental analysis were examined using electron microscopy and energy-dispersive X-ray spectroscopy (EDX). The morphology measurements were conducted using an Apreo+HiVac-scanning electron microscope (SEM, Thermo Fisher Scientific, Waltham, MA, USA) and a JEOL JEM-2100F (JEOL Ltd., Tokyo, Japan) transmission electron microscope (TEM). UV-Vis absorption spectra were recorded by a U-3900 visible spectrophotometer (Hitachi, Tokyo, Japan). The steady-state fluorescence spectra were obtained on an FL-4600 fluorescence spectrometer (Hitachi, Tokyo, Japan). The slit width was 5 and 5 nm, and the excitation wavelength was 480 nm. The infrared spectra (IR) were measured with a Nicolet+iS+50 Fourier transform infrared spectrometer (Thermo Fisher Scientific, Waltham, MA, USA). XRD patterns were recorded with a Bruker D8 Advance X-ray diffraction analyzer (Bruker AXS, Karlsruhe, Germany) using Cu-Kα radiation (λ = 1.5418 Å). Thermogravimetric analysis (TGA) was performed on an STA+449F5 TG-DTA analyzer (NETZSCH-Gerätebau GmbH, Bayern, Germany), and the heating process started from 20 °C at a ramp rate of 10 °C min^−1^ under air. The specific surface area was obtained by the Brunauer–Emmett–Teller (BET) calculation method. Pore size distributions were determined by the Barret–Joyner–Halenda (BJH) method from N_2_ adsorption–desorption isotherms, which were measured by accelerated surface area and porosimetry systems (Micromeritics ASAP 2460, Norcross, GA, USA).

### 2.3. Cell Lines Culture and Animal Experiments

Hela cells were obtained from American Type Culture Collection (ATCC), and then cultured in Dulbecco’s Modified Eagle Medium (DMEM) containing 10% FBS and 1% penicillin/streptomycin at 37 °C and under 5% CO_2_. Nude mice (18–22 g) were obtained from the Institute of Hematology & Hospital of Blood Disease, Chinese Academy of Medical Sciences & Peking Union Medical College (No. SCXK-2016-0006, Tianjin, China). The mice had free access to both solid rodent chow and clean water. The Institutional Animal Care Committee of Northwest University of Technology approved all experimental protocols involving animals in the work.

### 2.4. Preparation of H_2_L-MOFs

H_2_L-MOFs were synthesized with a simple solvothermal method. Zirconium tetrachloride (ZrCl_4_) (2.3 mg, 0.1 mmol) and H_2_L (8.8 mg, 0.1 mmol) were added in 10 mL DMF and 10 µL TFA, which was fully dissolved by ultrasound for 10 min. The mixed solution was then transferred into a Teflon-lined steel autoclave (Tianhe Keyan, Shandong, China) and heated at 100 °C for 72 h. The orange-red powder H_2_L-MOFs were obtained after centrifugation, washing thoroughly with DMF and ethanol, and drying at room temperature.

### 2.5. DOX Uptake Experiment

Doxorubicin (DOX) loading efficiency was determined by mixing H_2_L-MOFs (10 mg) and DOX•HCl (10 mg) in N-2-hydroxyethylpiperazine-N-ethane-sulphonicacid (HEPES) (0.1 M, 10 mL). After being stirred for 48 h at room temperature, the mixture of the solution was centrifugated at 12,000 rpm The supernatant solution was analyzed with a UV-vis spectrum at 480 nm, and the mass (m) of DOX in the solution was calculated according to the UV standard curve of DOX. The loading efficiency was calculated according to the following formula:

*E*_Load_ = (m_DOX_ − m)/m_MOF_ × 100% (1)

where m_DOX_ means the mass of DOX’s initial input (10 mg), and m_MOF_ means the mass of H_2_L-MOFs’ initial input (10 mg).

### 2.6. DOX Release Experiment

The DOX-loaded H_2_L-MOFs (H_2_L-MOFs@DOX) were put into an HEPES buffer (0.1 M) solution with a pH of 7.2 and an HAc-NaAc buffer (0.1 M) solution with a pH of 5.1 to imitate the environment in healthy tissue and in tumor tissue. After the drug released for 3, 6, 9,14, 24, 36 and 48 h, the amounts of released DOX in the solution were measured by UV-vis standard-curve spectrophotometry at 480 nm after centrifugation. The release efficiency was calculated based on the following equation:

*E*_Release_ = m_DOX_’/(m_DOX_) × 100% (2)

where m_DOX_’ means the mass of DOX released from H_2_L-MOFs@DOX and m_DOX_ means the mass of DOX loaded in H_2_L-MOFs.

### 2.7. In Vitro Singlet Oxygen Generation

The tumor microenvironment is characterized by a high level of hydrogen peroxide (H_2_O_2_) and weak acidity due to intense metabolic activities. H_2_O_2_ could react with a photosensitizer to produce singlet oxygen(^1^O_2_) [30,31]. The ^1^O_2_ generation of H_2_L-MOFs upon laser irradiation was conducted using an ABDA method. ^1^O_2_ generated from the H_2_L-MOFs oxidizes ABDA and decreases ABDA’s UV absorbance at 380 nm. In this study, the mixed solution of 80 μM H_2_L-MOFs (in saline) and 200 μM ABDA were irradiated with an LED lamp (light intensity 100 mW cm^−^^2^). The absorbance spectra of the solutions were recorded at each predetermined time interval. The stability of ABDA in PBS and H_2_L-MOFs in saline under irradiation were also tested as control experiments.

### 2.8. Cytotoxicity and In Vitro Chemotherapy, Photodynamic Therapy, and Chemo-Photodynamic Therapy of H_2_L-MOFs

The cytotoxicity of H_2_L-MOF nanoparticles was evaluated by the viability of Hela cells using a standard methyl thiazolyl tetrazolium (MTT) assay. Briefly, Hela cells were incubated into 96-well cell culture plates with a density of 5 × 10^3^ cells per well. Two groups of H_2_L-MOFs and H_2_L-MOFs@DOX were introduced to the medium at concentrations of 5, 15, 20, 40, and 80 µg/mL and incubated for 8 h after the Hela cells reached 90–95% confluence. After being washed three times in PBS solution, the photodynamic therapy (PDT) groups were irradiated with a blue-emission LED lamp (100 mW cm^−2^) for 10 min. Then, the plates were kept in an incubator under 37 °C and 5% CO_2_ for another 24 h. Then, MTT (3-(4,5-dimethyl-2-thiazolyl)-2,5-diphenyl-2-H-tetrazolium bromide) solution (20 μL 5.0 mg/mL, BBS) was successively added into each well. After 3 h, the remaining MTT solutions were removed. Then, in order to dissolve the formazan crystals, 150 μL dimethyl sulfoxide (Amresco, Albany, NY, USA) was added into each well. The absorbance of each well (OD value) was measured by a microplate reader (Promega, Beijing, China) at a wavelength of 550 nm. The cell viability was calculated as follows:

Cell viability % = (OD*_drug_*/OD*_control_*) × 100% (3)

### 2.9. In Vivo Fluorescence Imaging

In vivo fluorescence imaging was performed with nude mice as models. The mice were anesthetized with 4% chloral hydrate (6 mL kg^−1^). After the intravenous injection of H_2_L-MOFs solution (20 μmol Zr per kg), the fluorescence images were obtained by positioning the mice on the animal plate in the in vivo imaging system (NightOWL LB 983, Berthold Technologies GmbH & Co. KG, Bad Wildbad, Germany). The excitation and emission wavelengths were 480 and 646 nm, respectively. The data were obtained with IndiGO software (Vector, Stuttgart, Germany).

## 3. Results

### 3.1. Synthesis and Characterization of H_2_L-MOFs

**Figure 1:**
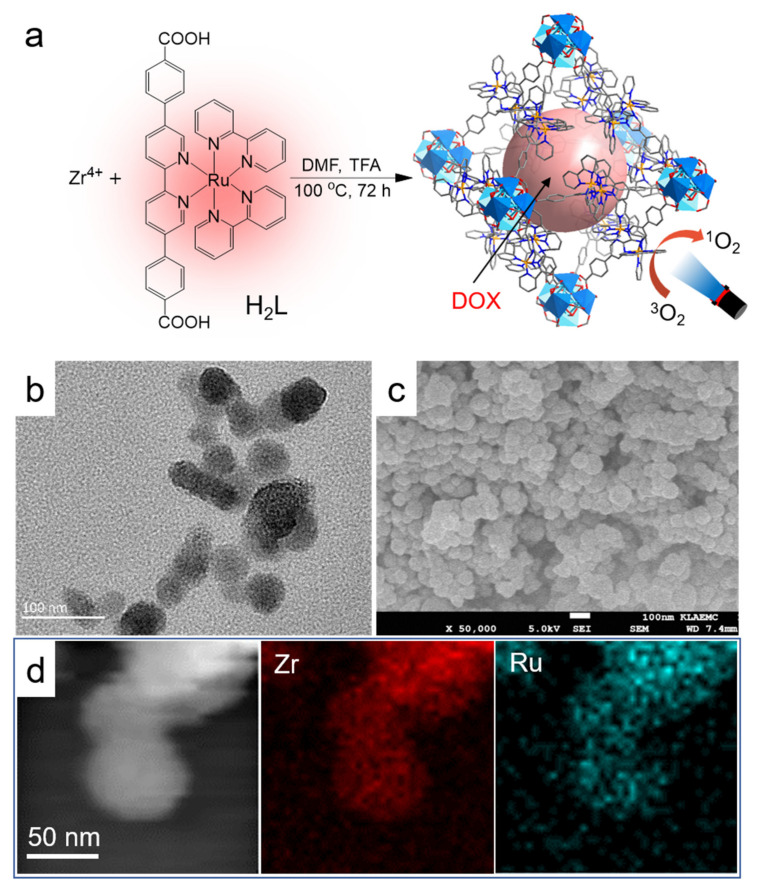
Structure and chemical composition analysis of H_2_L-MOF nanoparticles. (**a**) The schematic illustration of the synthesis of H_2_L-MOFs, uptake of drug for chemotherapy, and the processes of light-triggered singlet oxygen generation for PDT. (**b**)TEM image, (**c**) SEM image, and (**d**) STEM image and EDS elemental mapping of H_2_L-MOFs.

H_2_L and its derivatives have been used as ligands to construct bulk MOFs for catalysis application [32,33]. By slightly adjusting the reaction condition, we successfully prepared uniform H_2_L-MOF nanospheres by treating ZrCl_4_ and H_2_L in DMF and TFA (Figure 1a). The size and shape of MOF particles is important for their in vivo application [34]. By adjusting the ratio of DMF and TFA, monodisperse nanoparticles with an average diameter of 50 nm were achieved (Figure 1b,c and Figure S1). The nanosized H_2_L-MOFs showed good dispersion in water. The element mapping in Figure 1d and Figure S2 showed the uniform distribution of Zr and Ru elements in H_2_L-MOF nanoparticles. The powder X-ray diffraction (XRD) pattern (Figure S3) shows a significant amorphous component in H_2_L-MOFs, and the main peaks in 3.9 and 7.6 degrees for the crystalline component match the simulated pattern [32]. The wide peak at 20 degrees is mainly due to the scattering effect of H_2_L-MOFs nanoparticles. Discontinuous, anisotropic small lattices shown in the high-resolution transmission electron microscope (HRTEM) images (Figure S4) confirm that these MOF nanoparticles are partially crystallized. Both the HRTEM and XRD measurements illustrate that H_2_L-MOFs have short-ordered structures [35]. Thermogravimetric analysis (TGA) was used to investigate the thermal stability of the H_2_L-MOF (Figure S5). The weight loss of 5.5% in the range of 60–150 °C was attributed to the removal of the solvents’ DMF and water molecule. The structure remained stable up to 350 °C. EDS relative element content showed the rough content of C, N, and O elements, and also Ru and Zr metallic elements derived from the ligand H_2_L and metal node Zr^4+^, respectively. The porosity of H_2_L-MOFs was verified by N_2_ adsorption measurement (Figure S7). Nitrogen adsorption indicated that the Brunauer–Emmett–Teller (BET) surface area of the H_2_L-MOFs was 61.77 m^2^ g^−1^ (Figure S7a). H_2_L-MOFs exhibited a type IV N_2_ adsorption–desorption isotherm, which may have resulted from the existence of pores of different sizes. The not very high N_2_ uptake capacity may be attributed to the side chain of bipyridine ruthenium. Pore size distribution showed 1.8 nm and 3.1 nm pores (Figure S7b). However, the large pore size of H_2_L-MOFs is conducive to delivering molecules with large molecule sizes (Figure 1a).

### 3.2. Optical Properties of H_2_L-MOFs

**Figure 2:**
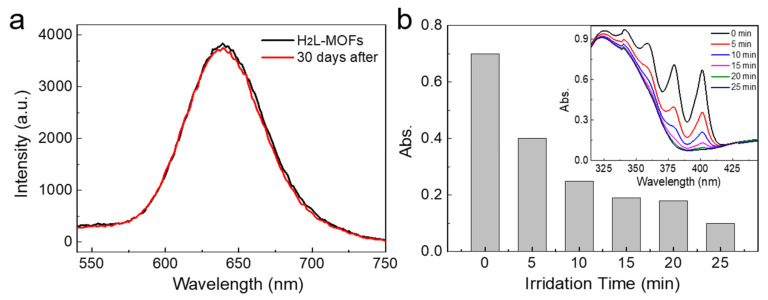
Optical properties of H_2_L-MOF nanoparticles. (**a**) The fluorescence spectrum of H_2_L-MOF nanoparticles right after the growth and 30 days after the growth. (**b**) The UV-vis absorbance intensity of ABDA at 380 nm after the addition of H_2_L-MOFs and under irradiation for different time periods. Inset: the corresponding UV-vis absorption spectra of the mixture of ABDA and H_2_L-MOFs under different irradiation time periods.

The optical properties of H_2_L-MOFs were investigated for their optical imaging and photodynamic therapy ability. As shown in Figure 2a, the H_2_L-MOFs showed a strong red emission at 646 nm in water, and the fluorescence spectrum remained almost unchanged after they were stored for 30 days at room temperature and with room light exposure. The red emission of the H_2_L-MOFs is advantageous to circumvent the disturbance from auto-fluorescence and avoid the scattering light of tissue, which facilitates realizing low background imaging [36]. The good emission stability illustrates the long-term stability of H_2_L-MOFs.

The derivatives of bipyridine ruthenium complex have been verified with excellent singlet oxygen (^1^O_2_) generation [30,31]. ABDA can be used as an indicator to determine the singlet oxygen generation, as it can react with singlet oxygen to decrease its absorbance at 380 nm [37,38]. The absorbance of the mixture of H_2_L-MOFs and ABDA attenuated 97% in only 20 min when irradiated with light (Figure 2b). Meanwhile, the absorbance of both H_2_L-MOFs and ABDA showed a high tolerance to the irradiation, illustrating that the attenuation is ascribed to the ^1^O_2_ produced by H_2_L-MOFs (Figure S8). The results illustrate the high ^1^O_2_ generation efficiency of H_2_L-MOFs and high PDT efficiency, which is higher than the analogues Ru[4,4′-(COOH)_2_bpy]_3_^2+^ based MOFs we reported before [35].

### 3.3. Drug Loading and Releasing of H_2_L-MOFs

**Figure 3:**
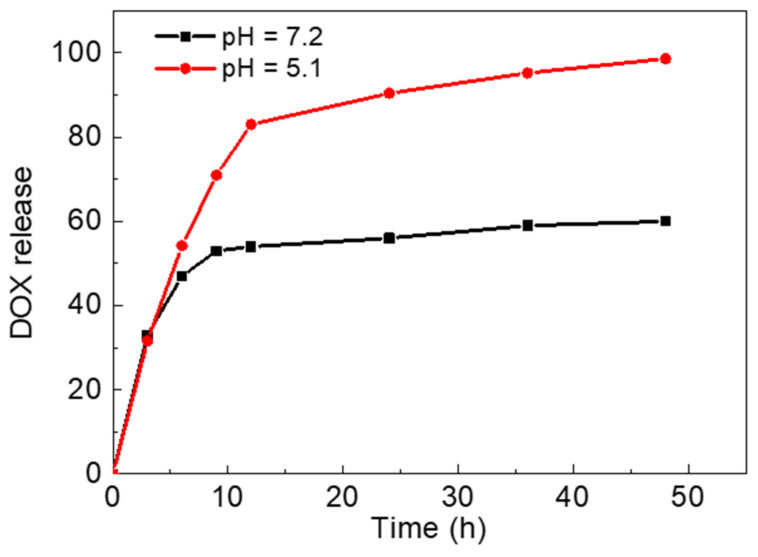
Release rate of DOX in buffer at pH = 5.1 and pH = 7.2.

Porous nanomaterials are excellent drug delivery carriers [10,12]. The large pores of H_2_L-MOFs make it a potential drug delivery carrier. Thus, we selected a commercialized anticancer drug, DOX, to investigate the drug adsorption and pH-responsive release properties of H_2_L-MOFs. The loading efficiency of DOX was verified to be as high as 94% after 48 h uptake (Figure S9). The high loading efficiency is attributed to the combination of electrostatic and noncovalent interactions, including π–π stacking effects and hydrophobic interactions [39]. In the simulated approximate normal physiological environment (pH 7.2), the release rate of DOX reached 53% in 10 h (Figure 3). In comparison, tumor cells tend to create an acidic extracellular environment, and in an acidic solution (pH 5.1) used to approximately simulate the tumor cell environment, the releasing rate reached 80% in 10 h. The controlled release of DOX may be attributed to the blocking buffer effect of the side chain bipyridine ruthenium group on the ligand H_2_L. The large side chain has both steric hindrance and weak hydrogen bonds with DOX from protecting DOX to burst release from the pore aperture of H_2_L-MOFs. Minati et al. reported that the polyelectrolyte complex layer coated mesoporous silica nanoparticles (MSN) for controlled drug release in cellular environment [12]. The work realized a delicately controlled drug release through the PAA/PAH complex layer. However, MSN usually has no imaging ability and can only be used as a drug delivery carrier alone. Thus, the pH-responsive release properties endow H_2_L-MOFs’ imaging nanoparticles with smart drug delivery capability for theranostics.

### 3.4. Biocompatibility of H_2_L-MOFs

**Figure 4:**
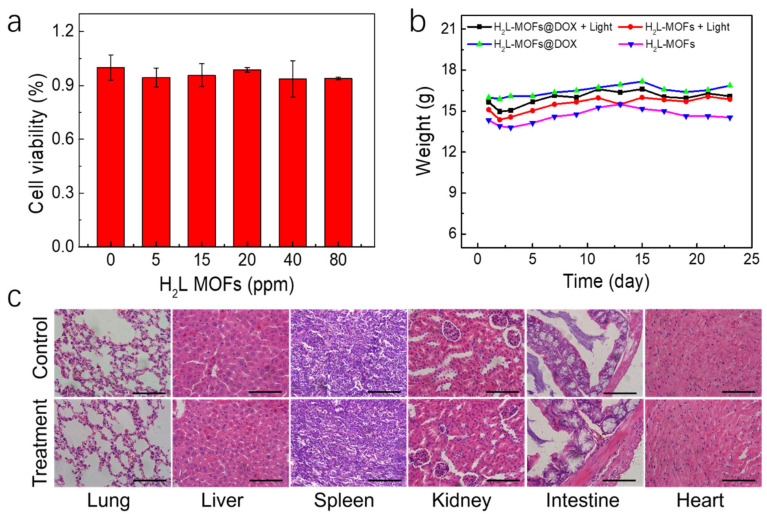
Biocompatibility study of H_2_L-MOF nanoparticles. (**a**) Viability of Hela cells incubated for 8 h in the presence of H_2_L-MOFs with different concentrations. (**b**) The body weight trends of the mice injected with H_2_L-MOFs and H_2_L- MOFs@DOX (20 μmol Zr per kg). (**c**) Histological images of the lung, liver, spleen, kidney, intestine, and heart of mice after injection with saline solution (control) and H_2_L-MOFs (treatment, 20 μmol Zr per kg). Scale bars: 100 μm.

The cytotoxicity of H_2_L-MOFs was tested with Hela cells using the standard 3-(4,5-dimethylthiazol-2-yl)-2,5-diphenyltetrazolium bromide (MTT) assay. The cell viability of Hela cells (a human cervical carcinoma cell line) was recorded after incubation with H_2_L-MOFs for 8 h (Figure 4a). The viability of cells did not decline after the incubation in the presence of 20 ppm H_2_L-MOFs (Figure 4a). More than 82% cell viability was observed with 80 ppm H_2_L-MOFs. Thus, H_2_L-MOFs are biocompatible and biosafe at the cellular level.

In vivo toxicity of H_2_L-MOFs was evaluated by growth observation of the main organs of the mice after the H_2_L-MOF treatment. After the intravenous injection of H_2_L-MOF nanoparticles, the weight and behavior of the mice were monitored and observed for a month. The weight of the mice increased during the following 4 weeks (Figure 4b), and none of the mice died. Meanwhile, histological changes in the main organs (liver, heart, spleen, intestine, kidney, and lung) of the mice two weeks after injection were examined to reveal the effect of H_2_L-MOF nanoparticles on the mice (Figure 4c). As shown in Figure 4c, no significant histological changes were observed between the control and the experimental groups, and no tissue damages were found upon H_2_L-MOFs injection. The in vivo results demonstrate that H_2_L-MOFs are highly biocompatible.

### 3.5. In Vitro Treatment of H_2_L-MOFs

**Figure 5:**
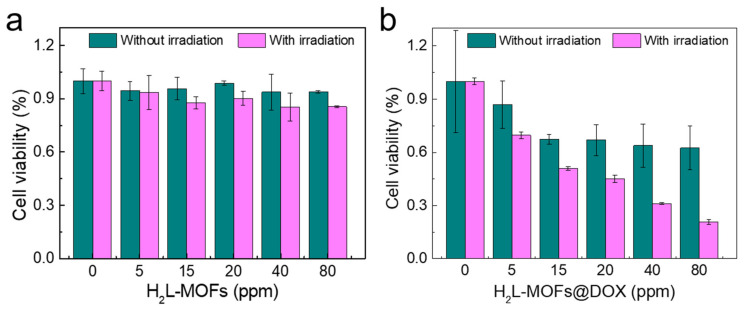
Cell viability of (**a**) H_2_L-MOFs and (**b**) H_2_L-MOFs@DOX.

The viability of Hela cells was measured to evaluate the in vitro PDT and DOX-release chemotherapy efficiency of H_2_L-MOFs using a standard MTT assay. The cell viability was recorded after incubation with H_2_L-MOFs for 8 h (Figure 5). The PDT-related groups were irradiated with a light of 480 nm for 10 min (0.1 W/cm^2^). The cell viability of the only H_2_L-MOFs-treated groups in all dosages was higher than 85%, indicating their high in vitro biocompatibility (Figure 5a blue). The viability of PDT-treated Hela cells was less than 85%, which confirmed the PDT efficiency of H_2_L-MOFs (Figure 5a pink). The smart delivery and release of DOX resulted in the chemotherapy group with a lower viability (70%) (Figure 5b blue). As a result, a low 50% inhibiting concentration (IC50) (20 ppm) and high cell lethality (80%) were observed following co-therapy of H_2_L-MOFs@DOX under irritation (Figure 5b pink). The high biocompatibility may be ascribed to the nanoscale of H_2_L-MOFs; the small size and approximate spherical morphology facilitate the successful uptake and metabolism [40]. The characteristics of easy uptake and metabolism further promote the PDT and chemotherapy efficiency.

### 3.6. In Vivo Fluorescence Imaging of H_2_L-MOFs

**Figure 6:**
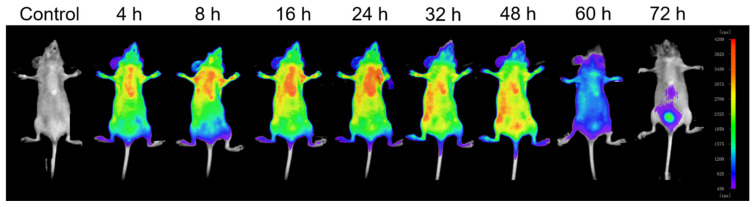
Fluorescence imaging of a normal nude mouse recorded before and after injection of H_2_L-MOFs.

The imaging efficiency and biodistribution of H_2_L-MOFs in mice were evaluated by real-time fluorescence imaging. After the intravenous injection of the H_2_L-MOFs into mice (20 μmol Zr per kg), the fluorescence signals were recorded instantly (Figure 6). Increased fluorescence signals of H_2_L-MOFs were observed in the liver in the first 24 h despite being acquired through intact skin and the skull. Then, the fluorescence signals transferred to the intestine from 24 to 48 h. Finally, the H_2_L-MOFs accumulated in the bladder from 48 to 72 h after injection (Figure 6). The H_2_L-MOFs were then observed to be excreted from the mice through urine. The fluorescence signal vanished about 84 h after the injection. The imaging results validated safe metabolism and the high sensitivity of the H_2_L-MOFs as a fluorescence imaging probe. The successful metabolism in mice also indicates the good biological safety of the material.

**Figure 7:**
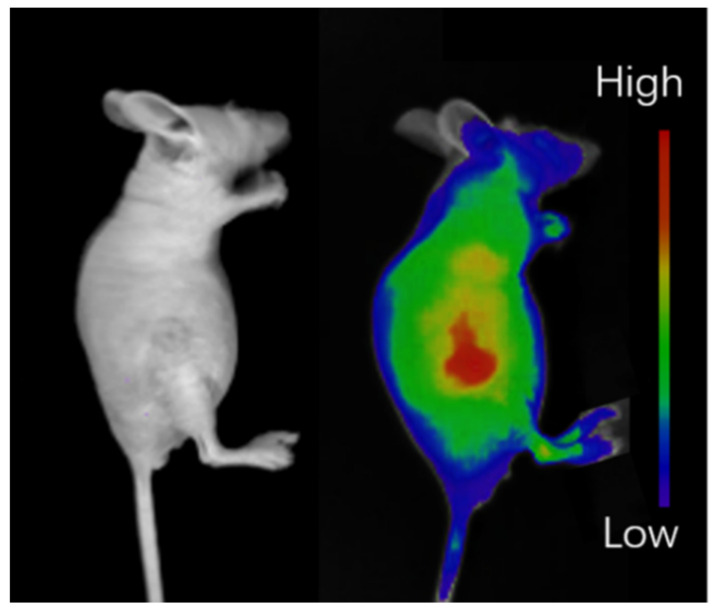
Fluorescence imaging of tumor sites in nude mice bearing tumors.

Fluorescence images with Hela tumor-bearing mice as models were recorded using H_2_L-MOF nanoparticles as imaging probes to demonstrate the in vivo tumor imaging diagnosis and imaging-guided therapy ability. The fluorescence signals of the H_2_L-MOFs appeared at the tumor site and liver of the mice because of the enhanced permeability and retention (EPR) effect [41,42] (Figure 7). The tumors were significantly distinguished from surrounding tissues, and the fluorescence intensity in the tumors maintained for 60 min (Figure 7). Thus, the tumor was clearly visualized with the fluorescence images, despite being acquired through intact skin and the skull. The fluorescence images of the dissected tumor validate the passive tumor targeting of H_2_L-MOFs (Figure S10). Thus, combining the good in vitro PDT and chemotherapy ability of H_2_L-MOFs, H_2_L-MOFs could be used for imaging diagnosis and imaging-guided therapy of tumors.

## 4. Conclusions

In summary, the biocompatible multifunctional H_2_L-MOFs were successfully developed by using zirconium ion and the long-molecule-structure ligand H_2_L as building units. Under single-wavelength excitation, simultaneous NIR-I fluorescence and singlet oxygen (^1^O_2_) generation were achieved, which showed the fluorescence imaging and photodynamic therapy potential. Moreover, the long-molecule structure of the ligand endows the H_2_L-MOFs with a large pore structure, which realized 94% DOX uptake. In vitro drug delivery tests further validated the drug delivery and sustained release. In vitro MTT cytotoxicity experiments showed efficient photodynamic therapy and chemotherapy through drug delivery. In vivo normal mice and tumor-bearing mice imaging validated the fluorescence imaging diagnosis capability of the H_2_L-MOFs. The report demonstrates a method for the convenient construction of theranostics probes.

## Data Availability

Data can be available upon request from the authors.

## Supplementary Materials

The following supporting information can be downloaded at: https://www.mdpi.com/article/10.3390/nano12020287/s1, Figure S1: Size distribution obtained from SEM images of H2L-MOFs; Figure S2. (**a**) SEM image and elemental mapping of (**b**) overlapped, (**c**) Zr, (**d**) Ru of the H2L-MOFs; Figure S3. XRD of H2L-MOFs; Figure S4. High Resolution Transmission Electron Microscope (HRTEM) image of H2L-MOFs nanoparticles; Figure S5. TGA plots of H2L-MOFs; Figure S6. EDS spectrum of H2L-MOFs; Figure S7. N2 adsorption isotherms for H2L-MOFs at 77 K. (**a**) N2 adsorption isotherms and (**b**) DFT pore size distribution for H2L-MOFs using data measured with N2 at 77 K; Figure S8. Control groups of (**a**) 200 μM ABDA and (**b**) 80 μM H2L-MOFs with the irradiation over a period of 25 min; Figure S9. DOX adsorption of MOFs. (**a**) Absorbance of DOX with different concentrations. (**b**) Standard curve of DOX solution. (**c**) DOX absorbance spectrum of H2L-MOFs after 48 h DOX adsorption; Figure S10. Fluorescence and intensity diagrams of various organs in nude mice bearing tumor. (**a**) stomach, lung, tumor, and intestine. (**b**) spleen, liver, kidney, and heart; Table S1. Relative element content obtained by EDS.
