# Supplementary material for: Single Nano-Sized Metal–Organic Framework for Bio-Nanoarchitectonics with In Vivo Fluorescence Imaging and Chemo-Photodynamic Therapy
Source: Nanomaterials (Basel). 2022 Jan 17;12(2):287. doi: 10.3390/nano12020287 (PMC8779747; doi:10.3390/nano12020287)
Supplement: Supplementary file 1 [file nanomaterials-12-00287-s001.zip › nanomaterials-1521623-supplementary.pdf]

nanomaterials

MDPI

Supplementary Information

Single Nano-Sized Metal–Organic Framework for Bio-Nanoarchitectonics with In Vivo Fluorescence Imaging and Chemo-Photodynamic Therapy

Yong-Mei Wang [1], Ying Xu [1], Xinxin Zhang [1], Yifan Cui [1], Qingquan Liang [1], Cunshun Liu [1], Xinan Wang [1], Shuqi Wu [2] and Rusen Yang [1,*]

[1] School of Advanced Materials and Nanotechnology, Academy of Advanced Interdisciplinary Research, Xidian University, Xi'an 710126, China; wangym1001@xidian.edu.cn (Y.-M.W.); 18141213000@stu.xidian.edu.cn (Y.X.); xxinzhang20@stu.xidian.edu.cn (X.Z.); 17170210019@stu.xidian.edu.cn (Y.C.); 21010540018@stu.xidian.edu.cn (Q.L.); 21141213493@stu.xidian.edu.cn (C.L.); wangxn@xidian.edu.cn (X.W.)
[2] School of Life Sciences, Northwest University of Technology, Xi'an 710072, China; sqwu@nwpu.edu.cn
* Correspondence: rsyang@xidian.edu.cn

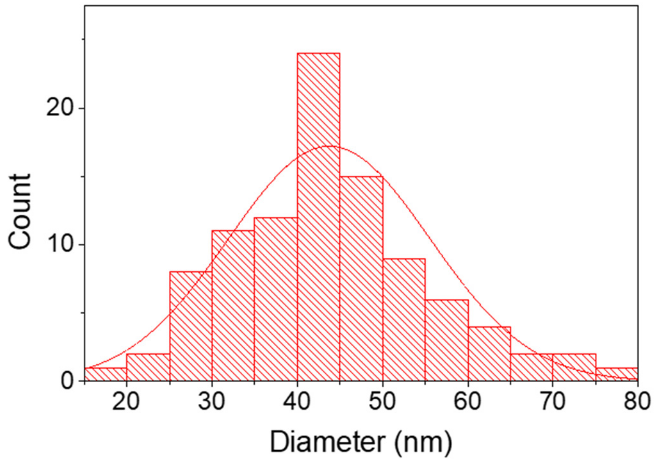

Figure S1. Size distribution obtained from SEM images of H_2L-MOFs.

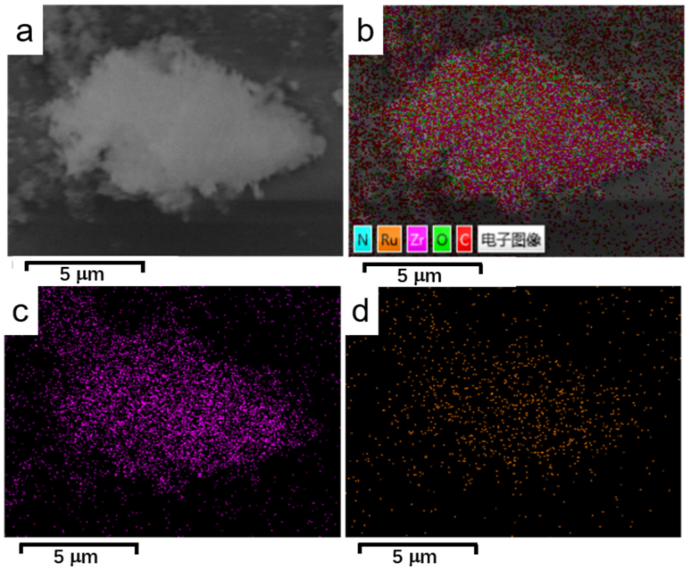

Figure S2. (**a**) SEM image and elemental mapping of (**b**) overlapped, (**c**) Zr, (**d**) Ru of the H_2L-MOFs.

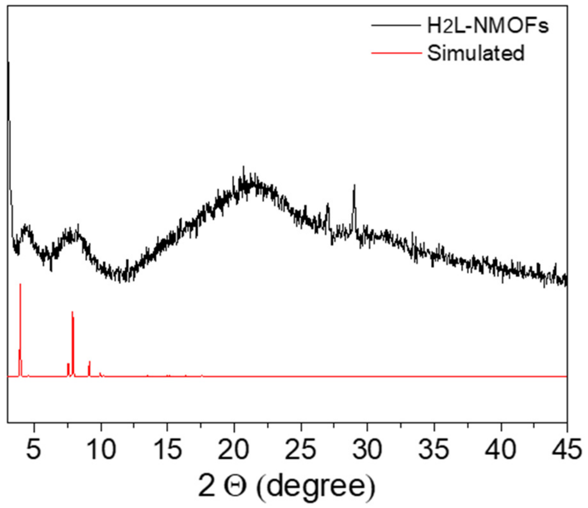

Figure S3. XRD of H_2L-MOFs.

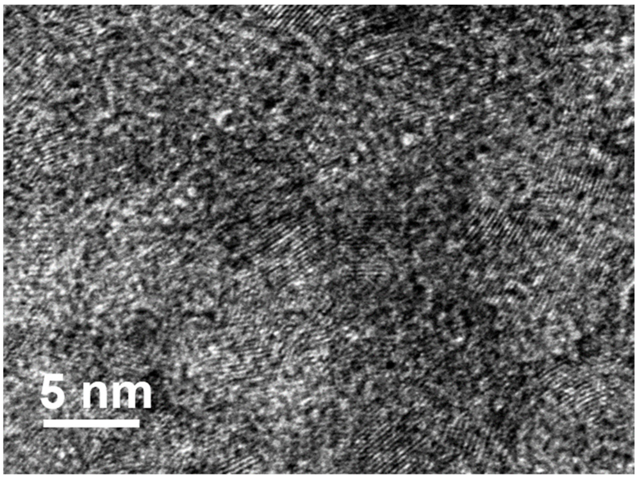

Figure S4. High Resolution Transmission Electron Microscope (HRTEM) image of H2L-MOFs nanoparticles.

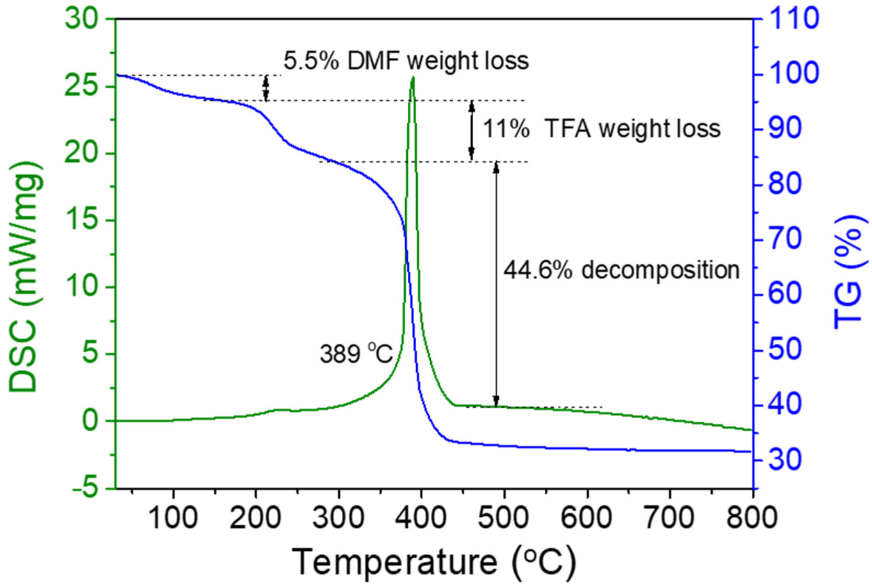

Figure S5. TGA plots of H_2L-MOFs.

Table S1. Relative element content obtained by EDS.

Element	Series	Apparent concentration	k ratio	wt%	wt% Sigma	Atomic percentage
C	K-series	30.54	0.30541	57.19	1.08	68.37
N	K-series	1.63	0.00290	2.61	1.49	2.67
O	K-series	17.47	0.05878	30.59	0.77	27.46
Zr	L-series	10.85	0.10855	8.67	0.34	1.36
Ru	L-series	1.07	0.01071	0.94	0.16	0.13
Total				100.00		100.00

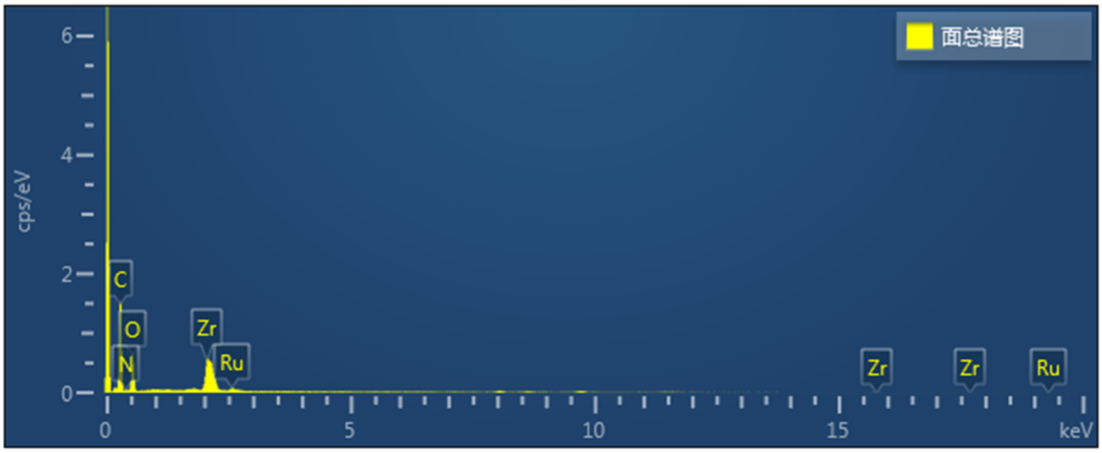

Figure S6. EDS spectrum of H_2L-MOFs.

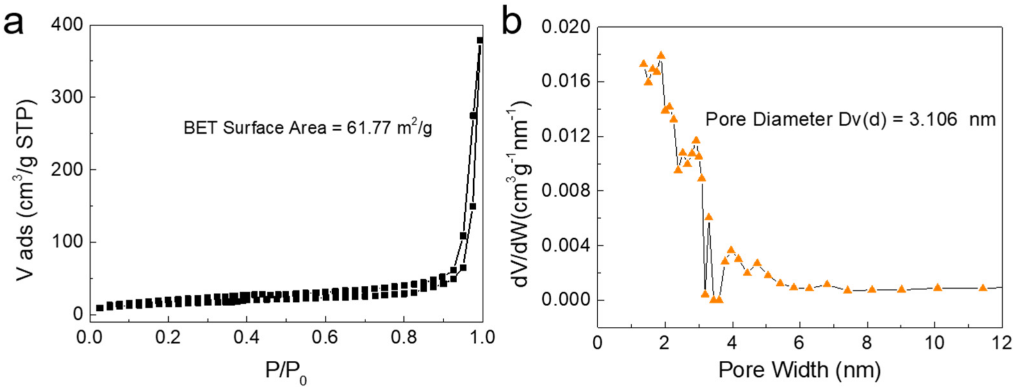

Figure S7. N_2 adsorption isotherms for H_2L-MOFs at 77 K. (**a**) N_2 adsorption isotherms and (**b**) DFT pore size distribution for H_2L-MOFs using data measured with N_2 at 77 K.

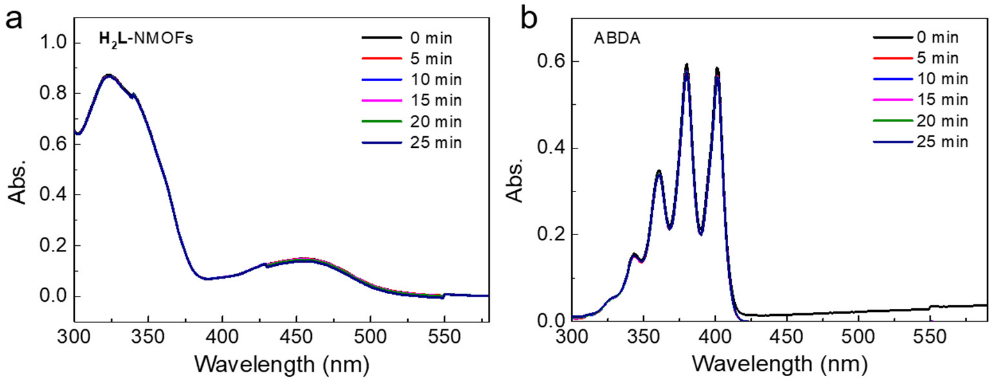

Figure S8. Control groups of (a) 200 μM ABDA and (b) 80 μM H_2L-MOFs with the irradiation over a period of 25 min.

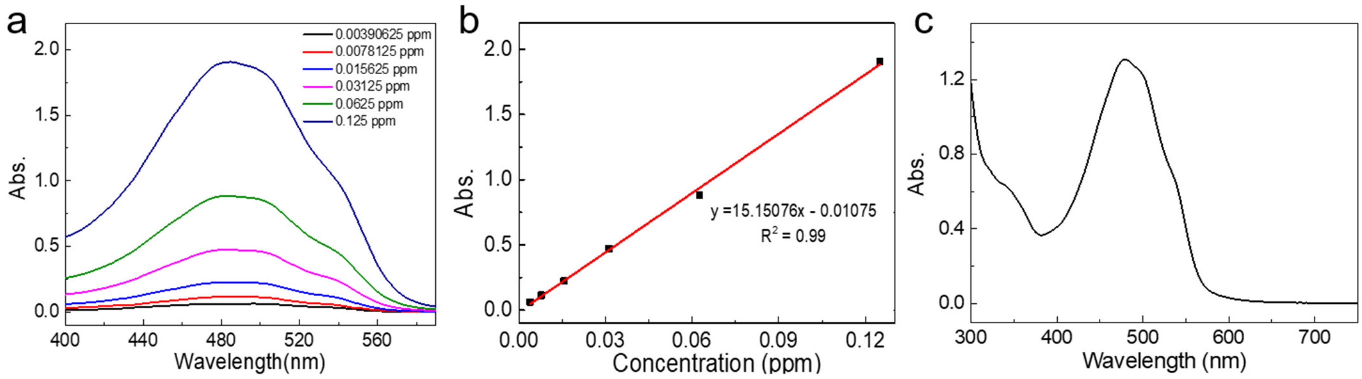

Figure S9. DOX adsorption of MOFs. (**a**) Absorbance of DOX with different concentrations. (**b**) Standard curve of DOX solution. (**c**) DOX absorbance spectrum of H_2L-MOFs after 48 h DOX adsorption.

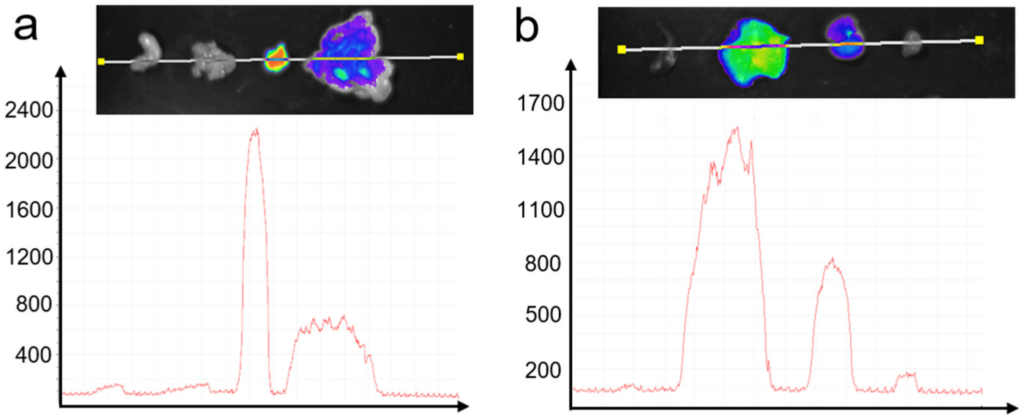

Figure S10. Fluorescence and intensity diagrams of various organs in nude mice bearing tumor. (**a**) stomach, lung, tumor, and intestine. (**b**) spleen, liver, kidney, and heart.
